# Impact of myocardial infarction status on long-term outcomes after PCI with the ultimaster biodegradable-polymer sirolimus-eluting stent

**DOI:** 10.3389/fcvm.2026.1903710

**Published:** 2026-10-30

**Authors:** Ki-Dong Yoo, Hun-Joo Lee, Sang-Suk Choi, Gyung-Min Park, Jin Jung, Seong-Hoon Lim, Sung-Ho Her, Jae-Hwan Lee, Seung Jin Lee, Jang-Whan Bae, Kyusup Lee

**Affiliations:** 1 Department of Cardiology, St. Vincent’s Hospital, The Catholic University of Korea, Suwon, Republic of Korea; 2 Catholic Research Institute for Intractable Cardiovascular Disease (CRID), College of Medicine, The Catholic University of Korea, Seoul, Republic of Korea; 3 Department of Cardiology, Daejeon St. Mary’s Hospital, College of Medicine, The Catholic University of Korea, Seoul, Republic of Korea; 4 Department of Cardiology in Internal Medicine, Chungnam National University Hospital, Daejeon, Republic of Korea; 5 Division of Cardiology, Department of Internal Medicine, Dankook University Hospital, Cheonan, Republic of Korea; 6 Department of Cardiology, Soonchunhyang University Cheonan Hospital, Cheonan-si, Chungcheongnam-do, Republic of Korea; 7 Department of Cardiology in Internal Medicine, Good Samsun Hospital, Busan, Republic of Korea; 8 Department of Cardiology, Ulsan University Hospital, University of Ulsan College of Medicine, Ulsan, Republic of Korea

**Keywords:** biodegradable-polymer sirolimus-eluting stent, landmark analysis, myocardial infarction, net adverse clinical events, percutaneous coronary intervention

## Abstract

**Background:**

Patients presenting with acute myocardial infarction (MI) represent a high-risk population after percutaneous coronary intervention (PCI), characterized by plaque instability, residual thrombus burden, and heightened ischemic and bleeding risk. However, the time-dependent risk profile and long-term durability of stent-related outcomes according to MI status have not been characterized for the Ultimaster^TM^ biodegradable-polymer sirolimus-eluting stent (BP-SES). We evaluated clinical outcomes according to MI status using a 1-month landmark approach.

**Methods:**

In this prospective, multicenter Korean registry, 932 patients who underwent PCI with the Ultimaster^TM^ or Ultimaster^TM^ Tansei^TM^ BP-SES were stratified into MI (*n* = 346) and non-MI (*n* = 586) groups. The primary endpoint was net adverse clinical events (NACE), a composite of cardiac death, MI, cerebrovascular accident, bleeding, and definite or probable stent thrombosis, assessed at 1 month, at 1 year, and from 1 month to 3 years using a 1-month landmark analysis.

**Results:**

At 1 month, NACE was significantly higher in the MI group [12.1% vs. 7.0%; hazard ratio [HR] 1.66, 95% confidence interval [CI] 1.01–2.72; *p* = 0.045], driven primarily by numerical excesses in cardiac death and bleeding. At 1 year, the cumulative incidence of NACE was numerically higher in the MI group (20.5% vs. 14.9%), a relative difference of approximately 40% without statistical significance, and 1-year target lesion failure was low and comparable (7.2% vs. 5.3%; *p* = 0.482). In the 1-month landmark analysis evaluating events from 1 month to 3 years, no significant differences were observed for any endpoint, including NACE (12.4% vs. 13.3%; HR 0.84, 95% CI 0.55–1.29; *p* = 0.425).

**Conclusion:**

The early excess of clinical events in MI patients undergoing PCI with the Ultimaster^TM^ BP-SES did not extend beyond the first month, and no significant differences in outcomes through 3 years were detected compared with non-MI patients. These findings suggest that, in the era of contemporary BP-SES and contemporary secondary prevention, MI status itself does not appear to be a major determinant of long-term outcomes after PCI, and are reassuring for the use of BP-SES across the spectrum of clinical presentations.

## Introduction

Percutaneous coronary intervention (PCI) with coronary stenting has become the cornerstone of treatment for obstructive coronary artery disease, and successive generations of drug-eluting stents (DES) have markedly improved clinical outcomes by reducing restenosis and target lesion revascularization (TLR) compared with bare-metal stents (BMS) (1). However, first-generation DES, which incorporated antiproliferative drugs within durable polymer coatings, were associated with delayed arterial healing, incomplete endothelial coverage, and persistent vascular inflammation, all of which contributed to an increased risk of late and very late stent thrombosis (2). To overcome these limitations, biodegradable polymer DES (BP-DES) were introduced as third-generation devices, in which the polymer coating is fully resorbed within several months after complete drug elution, leaving behind a bare-metal scaffold and promoting more physiological vascular healing while lowering the risk of late thrombotic events (3). Among contemporary BP-DES, the Ultimaster^TM^ and Ultimaster^TM^ Tansei^TM^ stents (Terumo, Tokyo, Japan) are thin-strut (80 μm), cobalt–chromium sirolimus-eluting stents featuring an abluminal-gradient bioresorbable poly(D,L-lactide-co-caprolactone) coating designed to fully degrade within 3 to 4 months, an architecture intended to combine effective neointimal suppression with favorable long-term vascular healing (4).

The safety and efficacy of the Ultimaster^TM^ stent have been evaluated across multiple randomized controlled trials and real-world registries. The CENTURY II trial demonstrated non-inferiority of Ultimaster^TM^ to durable polymer everolimus-eluting stents in a general population with coronary artery disease, with comparable rates of target lesion failure (TLF) sustained through 5 years of follow-up (4, 5). In real-world settings, the e-Ultimaster registry, which enrolled more than 36,000 patients, the ULISSE registry, and a large Japanese all-comers registry consistently reported low 1-year TLF rates of approximately 3 to 5%, supporting the safety and efficacy of Ultimaster^TM^ across diverse patient populations (6, 7). More recently, a Korean multicenter registry confirmed a 1-year TLF rate of 4.1% with low rates of stent thrombosis (0.6%), although follow-up was limited to 12 months and dedicated comparative analyses according to clinical presentation were not performed (8). Despite this accumulating evidence, data on the long-term safety and efficacy of the Ultimaster^TM^ and Ultimaster^TM^ Tansei^TM^ stents remain limited, particularly in patients presenting with acute myocardial infarction (MI), who represent a high-risk population characterized by plaque instability, residual thrombus burden, and a heightened propensity for both ischemic and bleeding events after PCI (9).

Importantly, adverse events after PCI in patients with acute MI are not distributed uniformly over time. Early events—occurring predominantly within the first 30 days—are largely driven by patient- and lesion-related factors such as hemodynamic instability, residual thrombus, and procedural complexity, whereas later events increasingly reflect stent-related processes including vascular healing and late thrombotic complications (9, 10). Conventional cumulative outcome analyses may therefore obscure stent-specific performance, particularly in the higher-risk MI group. To address this, landmark analysis has been increasingly used to separate these phase-specific effects and has provided important insights into the long-term safety of contemporary DES across different clinical presentations (10, 11). However, a dedicated comparison of MI vs. non-MI patients using a landmark approach has not been reported for the Ultimaster^TM^ biodegradable polymer sirolimus-eluting stent (BP-SES), leaving the time-dependent risk profile and the long-term durability of stent-related outcomes in MI patients incompletely defined.

Therefore, this study aimed to evaluate the clinical outcomes of patients undergoing PCI with the Ultimaster^TM^ and Ultimaster^TM^ Tansei^TM^ BP-SES according to MI status in a prospective, multicenter Korean registry, with a 1-month landmark analysis to assess net adverse clinical events (NACE) and individual endpoints at 1 month, at 1 year, and from 1 month to 3 years.

## Methods

### Study design and population

This study was a prospective, multicenter, observational registry conducted at eight cardiovascular centers in South Korea, all of which routinely perform PCI. Consecutive adult patients (≥19 years of age) who underwent PCI with implantation of at least one Ultimaster^TM^ or Ultimaster^TM^ Tansei^TM^ BP-SES (Terumo, Tokyo, Japan) for the treatment of coronary artery disease between June 27, 2016 and March 14, 2022 were eligible for inclusion. Patients were excluded if they met any of the following criteria: (1) concomitant implantation of a different DES or BMS during the index procedure; (2) anticipated life expectancy of less than 1 year due to a non-cardiac condition; or (3) presence of cardiogenic shock at the time of PCI.

The study protocol complied with the principles of the Declaration of Helsinki and was approved by the Institutional Review Board of St. Vincent's Hospital (IRB No. VC18OEDI0182) and the institutional review boards of all participating centers. All participants provided written informed consent prior to enrollment. For the present analysis, patients were classified according to their clinical presentation at the index procedure into two groups for comparison: the MI group (ST-elevation MI [STEMI] or non–ST-elevation MI [NSTEMI]) and the non-MI group (unstable angina or stable angina).

### Study device and PCI procedure

The Ultimaster^TM^ and Ultimaster^TM^ Tansei^TM^ BP-SES (Terumo, Tokyo, Japan) are thin-strut (80 μm) cobalt–chromium coronary stents featuring an abluminal-gradient bioresorbable poly(D,L-lactide-co-caprolactone) coating that elutes sirolimus. The polymer is designed to fully resorb within 3 to 4 months after implantation, leaving behind a bare-metal scaffold and supporting early vascular healing. The Ultimaster^TM^ Tansei^TM^ shares the same stent platform, polymer, and drug formulation as the Ultimaster^TM^ and differs only in its delivery catheter, which is engineered for enhanced trackability.

All PCI procedures were performed according to contemporary guideline-recommended practices. Decisions regarding lesion preparation, stent sizing, the use of intravascular imaging, adjunctive plaque modification techniques such as rotational atherectomy, post-dilation, and the use of glycoprotein IIb/IIIa inhibitors or mechanical circulatory support devices were made by the treating interventional cardiologist on a per-case basis. Periprocedural antithrombotic therapy was administered in accordance with institutional standards.

After the procedure, all patients received guideline-directed medical therapy individualized to each patient's clinical profile, with the duration of dual antiplatelet therapy determined at the discretion of the treating physician.

### Study endpoints and definitions

The primary endpoint of this study was NACE, defined as a composite of cardiac death (CD), MI, cerebrovascular accident (CVA), bleeding, and definite or probable stent thrombosis (ST). The primary endpoint and its components were assessed at three time frames: at 1 month, at 1 year, and from 1 month to 3 years using a 1-month landmark analysis.

Secondary endpoints included the individual components of NACE, repeat revascularization (RR), target vessel revascularization (TVR), TLR, target vessel failure (TVF), and TLF. TLF was defined as a composite of CD, target vessel MI, or TLR. TVF was defined as a composite of CD, target vessel MI, or TVR.

All clinical events were adjudicated according to the following standardized definitions. MI was defined according to the Fourth Universal Definition of Myocardial Infarction (12). CD was defined as any death due to a cardiac cause (e.g., MI, low-output heart failure, fatal arrhythmia), as well as unwitnessed death, death of unknown cause, and all procedure-related deaths. CVA was defined as a stroke (ischemic or hemorrhagic), transient ischemic attack, or reversible ischemic neurologic deficit, confirmed by neurological evaluation and/or imaging. Bleeding events were classified as major or minor according to a modified Thrombolysis in Myocardial Infarction (TIMI) criteria (13). Major bleeding encompassed fatal bleeding, intracranial, intraocular, or retroperitoneal hemorrhage, hemodynamically significant bleeding requiring intervention, bleeding requiring transfusion of one or more units of packed red cells or whole blood, or a hemoglobin drop of ≥3 g/dL. Minor bleeding comprised clinically overt bleeding not meeting major criteria, including gross hematuria unrelated to trauma, epistaxis, gastrointestinal bleeding, hemoptysis, subconjunctival hemorrhage, a hematoma larger than 5 cm or requiring hospitalization, and bleeding associated with a hemoglobin drop of 2 to 3 g/dL. Total bleeding was defined as the occurrence of either major or minor bleeding. ST was defined according to the Academic Research Consortium (ARC) criteria as definite or probable (14).

All suspected endpoint events were independently reviewed and adjudicated by a Clinical Events Committee composed of cardiologists who were not directly involved in the index PCI. Adjudication was performed centrally based on source documentation provided by the participating sites.

### Data collection and follow-up

Baseline demographic and clinical characteristics, cardiovascular risk factors, comorbidities, laboratory values, procedural and angiographic details, and discharge medications were collected at the index hospitalization. All data were entered into a web-based electronic case report form (e-CRF) using anonymized subject identifiers. Data integrity and protocol adherence were monitored centrally by the Data Coordinating Center at Daejeon St. Mary's Hospital.

Clinical follow-up was scheduled at 1, 6, and 12 months after the index procedure, and annually thereafter up to 3 years. Follow-up data were obtained through outpatient clinic visits, structured telephone interviews, and review of medical records.

### Statistical analysis

Continuous variables are presented as mean ± standard deviation and were compared between groups using the Student's t-test or the Wilcoxon rank-sum test, as appropriate. Categorical variables are expressed as counts and percentages and were compared using the chi-square test or Fisher's exact test.

Cumulative event rates were estimated using the Kaplan–Meier method, and between-group comparisons were performed with the log-rank test. A 1-month landmark analysis was conducted to evaluate clinical outcomes from 1 month to 3 years among patients who were event-free at 1 month, in order to separate early non–stent-related events from later stent-related outcomes. To formally assess whether the association between MI status and each outcome differed between the early (within 1 month) and later (1 month to 3 years) periods, a multivariable Cox model including an interaction term between MI status and follow-up period was fitted, and the *P* value for interaction was reported. Hazard ratios (HRs) and 95% confidence intervals (CIs) were calculated using univariable and multivariable Cox proportional hazards models. The multivariable model included age, sex, hypertension, diabetes mellitus, current smoker, previous PCI, family history of CAD, creatinine, LVEF, ACC/AHA lesion classification, and IVUS use as covariates; the same covariates were used for all endpoints and time frames. Unless otherwise specified, HRs cited in the text are from the multivariable models.

For patients lost to follow-up, data were censored at the date of last contact. All statistical analyses were performed using SAS software, version 9.4 (SAS Institute, Cary, NC, USA). A two-sided *p* value < 0.05 was considered statistically significant.

## Results

### Patient population, baseline, and procedural characteristics

Of 1,001 patients enrolled at the participating centers, 69 were excluded before group classification because their clinical presentation (MI status) could not be determined, owing to missing procedural angiographic data in 4 patients and missing clinical data in 65 patients, leaving 932 patients in the final analysis. The final study population comprised 346 patients in the MI group (STEMI, *n* = 188; NSTEMI, *n* = 158) and 586 patients in the non-MI group (unstable angina, *n* = 376; stable angina, *n* = 210) (Figure 1). Follow-up at 1 year was available in 888 patients (95.3%; 565 of 586 non-MI and 323 of 346 MI patients), and 3-year follow-up was completed in 858 patients (92.1%; 551 non-MI and 307 MI patients). The median follow-up duration was 35.9 months (interquartile range 34.4–36.9). Within the first month, 83 patients experienced a NACE and 2 patients were censored without an event, and the remaining 847 patients who were event-free at 1 month entered the landmark analysis for the primary endpoint.

**Figure 1:**
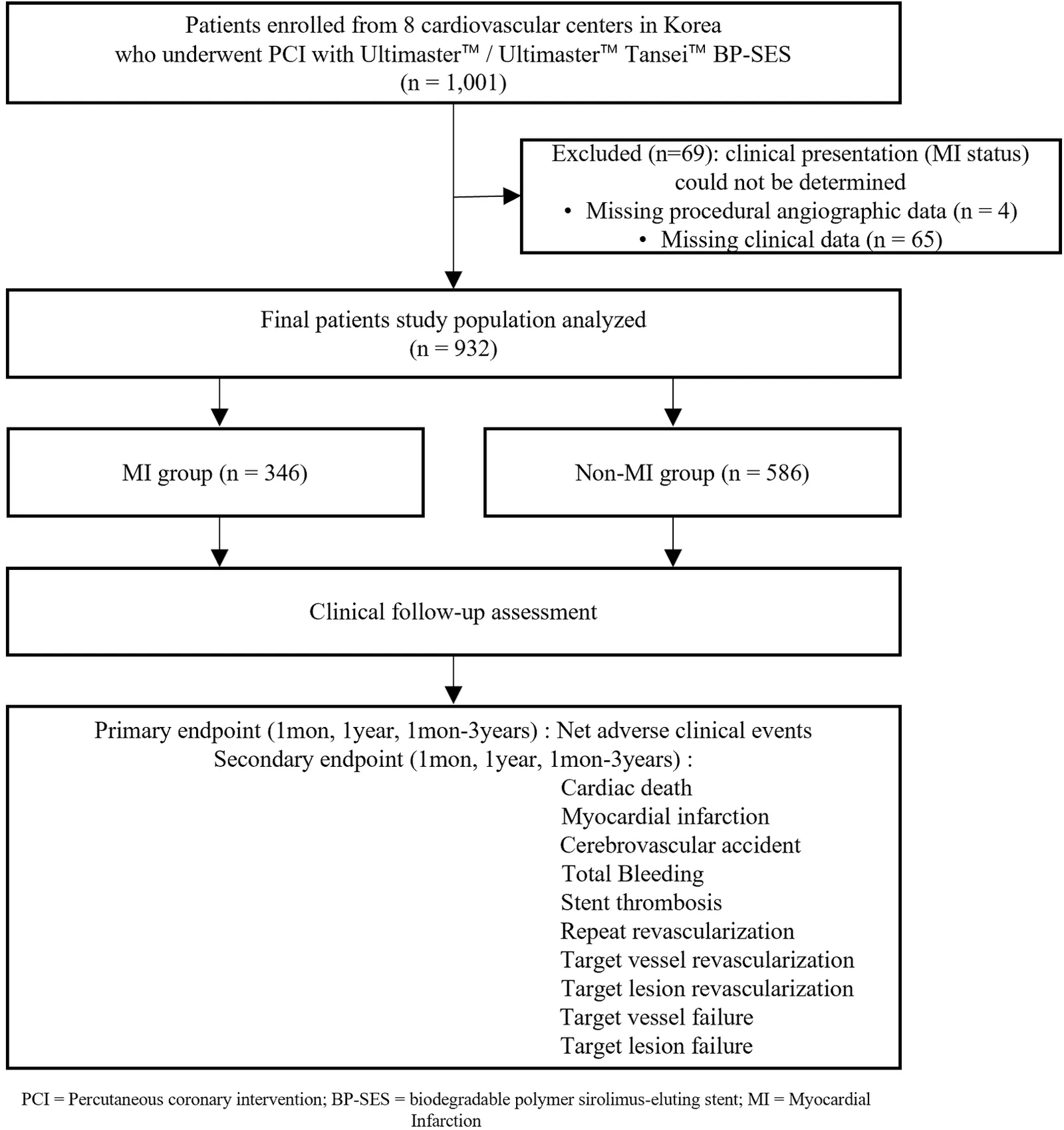
Study flow diagram. Study flow diagram of the prospective, multicenter Korean registry. Of 1,001 patients enrolled at 8 cardiovascular centers who underwent PCI with the Ultimaster^TM^ or Ultimaster^TM^ Tansei^TM^ BP-SES, 69 were excluded before group classification because their clinical presentation (MI status) could not be determined (missing clinical data, *n* = 65; missing procedural angiographic data, *n* = 4), leaving 932 patients in the final analytic cohort. Patients were stratified into the MI group (*n* = 346) and the non-MI group (*n* = 586) and underwent clinical follow-up assessment for the primary and secondary endpoints at 1 month, 1 year, and from 1 month to 3 years. PCI = percutaneous coronary intervention; BP-SES = biodegradable polymer sirolimus-eluting stent; MI = myocardial infarction.

Baseline demographic and clinical characteristics of the two groups are shown in Table 1. Compared with non-MI patients, those in the MI group were significantly younger (65.4 ± 12.6 vs. 68.9 ± 10.8 years, *p* < 0.001) and more often male (71.4% vs. 63.0%, *p* = 0.009) or current smokers (34.4% vs. 19.1%, *p* < 0.001), and they more frequently had a family history of coronary artery disease (6.4% vs. 2.9%, *p* = 0.011). In contrast, hypertension (51.7% vs. 68.3%), diabetes mellitus (28.3% vs. 36.7%), and previous PCI (9.8% vs. 17.9%) were less prevalent in the MI group (all *p* ≤ 0.009). Total cholesterol and low-density lipoprotein cholesterol (LDL-C) levels were higher in the MI group (179.0 ± 44.8 vs. 155.2 ± 42.7 mg/dL, and 117.9 ± 43.6 vs. 100.2 ± 64.0 mg/dL; both *p* < 0.001), and left ventricular ejection fraction was lower (52.0 ± 12.2% vs. 60.3 ± 11.3%, *p* < 0.001). Discharge medications also differed in line with clinical presentation, with ticagrelor and beta-blockers more frequently prescribed in the MI group, and clopidogrel more commonly used in the non-MI group.

**Table 1:** Baseline demographic and clinical characteristics.

Variable	Total (*n* = 932)	Non-MI (*n* = 586)	MI (*n* = 346)	*p*-value
Age, years	67.6 ± 11.7	68.9 ± 10.8	65.4 ± 12.6	<0.001
Male sex	616 (66.1)	369 (63.0)	247 (71.4)	0.009
Current smoker	231 (24.8)	112 (19.1)	119 (34.4)	<0.001
BMI, kg/m^2^	24.6 ± 3.7	24.9 ± 3.8	24.1 ± 3.6	0.001
Clinical diagnosis				
Stable angina	210 (22.5)	210 (35.8)	0 (0.0)	<0.001
Unstable angina	376 (40.3)	376 (64.2)	0 (0.0)	<0.001
NSTEMI	158 (17.0)	0 (0.0)	158 (45.7)	<0.001
STEMI	188 (20.2)	0 (0.0)	188 (54.3)	<0.001
Hypertension	579 (62.1)	400 (68.3)	179 (51.7)	<0.001
Diabetes mellitus	313 (33.6)	215 (36.7)	98 (28.3)	0.009
Dyslipidemia	645 (69.2)	402 (68.6)	243 (70.2)	0.602
Previous CABG	10 (1.1)	8 (1.4)	2 (0.6)	0.260
Previous MI	62 (6.7)	42 (7.2)	20 (5.8)	0.412
Previous PCI	139 (14.9)	105 (17.9)	34 (9.8)	0.001
Family history of CAD	39 (4.2)	17 (2.9)	22 (6.4)	0.011
Previous HF	21 (2.3)	11 (1.9)	10 (2.9)	0.314
Previous CVA	99 (10.6)	69 (11.8)	30 (8.7)	0.137
Previous PVD	42 (4.5)	32 (5.5)	10 (2.9)	0.068
LVEF, %	57.2 ± 12.3	60.3 ± 11.3	52.0 ± 12.2	<0.001
Laboratory findings				
Hemoglobin, g/dL	13.4 ± 1.9	13.1 ± 1.9	13.8 ± 2.0	<0.001
Creatinine, mg/dL	1.4 ± 1.9	1.4 ± 2.0	1.3 ± 1.7	0.741
Total cholesterol, mg/dL	164.0 ± 45.0	155.2 ± 42.7	179.0 ± 44.8	<0.001
Triglyceride, mg/dL	139.6 ± 91.7	138.6 ± 88.3	141.3 ± 97.8	0.694
HDL cholesterol, mg/dL	43.9 ± 12.2	43.8 ± 11.9	44.0 ± 12.8	0.873
LDL cholesterol, mg/dL	106.2 ± 58.4	100.2 ± 64.0	117.9 ± 43.6	<0.001
hs-CRP, mg/dL	2.8 ± 39.5	3.5 ± 47.5	1.2 ± 2.7	0.464
HbA1c, %	6.4 ± 1.4	6.4 ± 1.3	6.5 ± 1.5	0.157
Discharge medications				
Aspirin	896 (96.1)	557 (95.1)	339 (98.0)	0.025
Clopidogrel	619 (66.4)	505 (86.2)	114 (33.0)	<0.001
Ticagrelor	232 (24.9)	36 (6.1)	196 (56.7)	<0.001
Prasugrel	68 (7.3)	36 (6.1)	32 (9.3)	0.078
Beta-blocker	529 (56.8)	291 (49.7)	238 (68.8)	<0.001
ACEi or ARB	480 (51.5)	288 (49.2)	192 (55.5)	0.061
Statin	867 (93.0)	537 (91.6)	330 (95.4)	0.030
NOAC	66 (7.1)	42 (7.2)	24 (6.9)	0.894

Values are mean ± standard deviation or n (%).

BMI, body mass index; NSTEMI, non–ST-segment elevation myocardial infarction; STEMI, ST-segment elevation myocardial infarction; CABG, coronary artery bypass grafting; MI, myocardial infarction; PCI, percutaneous coronary intervention; CAD, coronary artery disease; HF, heart failure; CVA, cerebrovascular accident; PVD, peripheral vascular disease; LVEF, left ventricular ejection fraction; HDL, high-density lipoprotein; LDL, low-density lipoprotein; hs-CRP, high-sensitivity C-reactive protein; HbA1c, hemoglobin A1c; ACE, angiotensin-converting enzyme; ACEi, angiotensin-converting enzyme inhibitor; ARB, angiotensin II receptor blocker; NOAC, non–vitamin K antagonist oral anticoagulant.

Angiographic and procedural characteristics are presented in Table 2. Type C lesions according to the American College of Cardiology/American Heart Association (ACC/AHA) classification were more frequent in the MI group (86.7% vs. 68.3%, *p* < 0.001), whereas the use of intravascular ultrasound was higher in the non-MI group (48.1% vs. 34.7%, *p* < 0.001). The two groups did not differ significantly in heavy calcification, target vessel distribution, prevalence of left main or multivessel disease, mean stent diameter, total number of stents, total stent length, or the presence of bifurcation lesions.

**Table 2:** Angiographic and procedural characteristics.

Variable	Total (*n* = 932)	Non-MI (*n* = 586)	MI (*n* = 346)	*p*-value
ACC/AHA lesion classification				
A	11 (1.2)	7 (1.2)	4 (1.2)	<0.001
B1	125 (13.4)	110 (18.8)	15 (4.3)	<0.001
B2	96 (10.3)	69 (11.8)	27 (7.8)	<0.001
C	700 (75.1)	400 (68.3)	300 (86.7)	<0.001
Heavy calcification	103 (11.1)	67 (11.4)	36 (10.4)	0.628
Left main treated	51 (5.5)	36 (6.1)	15 (4.3)	0.241
Multivessel disease	492 (52.8)	301 (51.4)	191 (55.2)	0.257
IVUS use	402 (43.1)	282 (48.1)	120 (34.7)	<0.001
Target vessel				
LAD treated	571 (61.3)	366 (62.5)	205 (59.3)	0.331
LCx treated	285 (30.6)	178 (30.4)	107 (30.9)	0.860
RCA treated	373 (40.0)	221 (37.7)	152 (43.9)	0.061
Ramus treated	9 (1.0)	6 (1.0)	3 (0.9)	0.813
Stent characteristics				
Mean stent diameter, mm	3.2 ± 0.4	3.2 ± 0.4	3.2 ± 0.5	0.102
Total number of stents	1.6 ± 0.9	1.6 ± 0.9	1.6 ± 0.9	0.944
Total stent length, mm	40.0 ± 26.9	40.1 ± 27.3	40.0 ± 26.3	0.954
Bifurcation lesion	107 (11.5)	65 (11.1)	42 (12.1)	0.628

Values are mean ± standard deviation or n (%).

ACC, American College of Cardiology; AHA, American Heart Association; IVUS, intravascular ultrasound; LAD, left anterior descending artery; LCx, left circumflex artery; RCA, right coronary artery; MI, myocardial infarction.

### Clinical outcomes

At 1 month after PCI, the incidence of NACE was significantly higher in the MI group than in the non-MI group (12.1% vs. 7.0%; HR 1.66, 95% CI 1.01–2.72; *p* = 0.045) (Table 3). Bleeding tended to occur more frequently in the MI group (9.5% vs. 6.1%; HR 1.60, 95% CI 0.93–2.74; *p* = 0.088), and a numerical excess in CD was also observed, although neither difference reached statistical significance. The remaining individual endpoints did not differ significantly between the two groups at 1 month.

**Table 3:** Clinical outcomes at 1 month and during the 1-month landmark period in MI versus non-MI patients.

Outcome	Total (*n* = 932)	Non-MI (*n* = 586)	MI (*n* = 346)	Log-rank *p*-value	Univariable HR (95% CI)	*p*-value	Multivariable HR (95% CI)	*p*-value	P for interaction
At 1 month									
CD	7 (0.8)	2 (0.3)	5 (1.5)	0.070	4.06 (0.79–20.95)	0.094	1.05 (0.12–9.20)	0.964	
MI	5 (0.5)	4 (0.7)	1 (0.3)	0.395	0.40 (0.04–3.57)	0.411	0.38 (0.04–4.23)	0.433	
CVA	2 (0.2)	0 (0.0)	2 (0.6)	0.082	—	—	—	—	
Total Bleeding	69 (7.4)	36 (6.1)	33 (9.5)	0.069	1.53 (0.96–2.46)	0.075	1.60 (0.93–2.74)	0.088	
Major bleeding	3 (0.3)	2 (0.3)	1 (0.3)	0.896	–	–	–	–	–
Minor bleeding	66 (7.1)	34 (5.8)	32 (9.3)	0.060	–	–	–	–	–
ST	3 (0.3)	1 (0.2)	2 (0.6)	0.295	3.34 (0.30–36.85)	0.325	4.48 (0.34–58.04)	0.252	
RR	5 (0.5)	2 (0.3)	3 (0.9)	0.316	2.43 (0.41–14.54)	0.332	4.22 (0.61–29.32)	0.145	
TVR	4 (0.4)	1 (0.2)	3 (0.9)	0.119	5.04 (0.52–48.40)	0.162	9.55 (0.87–104.66)	0.065	
TLR	4 (0.4)	1 (0.2)	3 (0.9)	0.119	5.04 (0.52–48.40)	0.162	9.55 (0.87–104.66)	0.065	
TVF	15 (1.6)	6 (1.0)	9 (2.6)	0.090	2.38 (0.84–6.68)	0.101	1.74 (0.49–6.18)	0.393	
TLF	15 (1.6)	6 (1.0)	9 (2.6)	0.090	2.38 (0.84–6.68)	0.101	1.74 (0.49–6.18)	0.393	
NACE	83 (8.9)	41 (7.0)	42 (12.1)	0.017	1.66 (1.08–2.56)	0.020	1.66 (1.01–2.72)	0.045	
From 1 month to 3 years (landmark analysis)									
CD	45 (4.8)	25 (4.3)	20 (5.8)	0.235	1.43 (0.79–2.57)	0.238	1.20 (0.61–2.37)	0.596	0.987
MI	5 (0.5)	3 (0.5)	2 (0.6)	0.927	1.09 (0.18–6.51)	0.927	0.87 (0.11–6.65)	0.891	0.777
CVA	19 (2.0)	14 (2.4)	5 (1.5)	0.397	0.65 (0.23–1.79)	0.401	0.52 (0.17–1.64)	0.265	0.986
Total Bleeding	81 (8.7)	52 (8.9)	29 (8.4)	0.893	1.03 (0.66–1.62)	0.893	1.01 (0.60–1.71)	0.960	0.440
Major bleeding	6 (0.6)	4 (0.7)	2 (0.6)	0.922	–	–	–	–	–
Minor bleeding	75 (8.0)	48 (8.2)	27 (7.8)	0.867	–	–	–	–	–
ST	3 (0.3)	2 (0.3)	1 (0.3)	0.917	0.88 (0.08–9.71)	0.917	1.74 (0.06–49.76)	0.745	0.998
RR	99 (10.6)	64 (10.9)	35 (10.1)	0.935	0.98 (0.65–1.48)	0.935	0.83 (0.52–1.33)	0.436	0.159
TVR	48 (5.2)	32 (5.5)	16 (4.6)	0.714	0.89 (0.49–1.63)	0.714	0.83 (0.42–1.63)	0.585	0.075
TLR	37 (4.0)	25 (4.3)	12 (3.5)	0.667	0.86 (0.43–1.71)	0.667	0.61 (0.28–1.32)	0.209	0.066
TVF	91 (9.8)	56 (9.6)	35 (10.1)	0.882	1.03 (0.68–1.57)	0.882	0.94 (0.58–1.51)	0.784	0.703
TLF	82 (8.8)	50 (8.5)	32 (9.3)	0.809	1.06 (0.68–1.65)	0.808	0.85 (0.51–1.41)	0.530	0.878
NACE	121 (13.0)	78 (13.3)	43 (12.4)	0.818	0.96 (0.66–1.39)	0.819	0.84 (0.55–1.29)	0.425	0.588

Values are n (%).

Hazard ratios (HRs) and 95% confidence intervals (CIs) were calculated using univariable and multivariable Cox proportional hazards models, with the non-MI group as the reference. The multivariable model included age, sex, hypertension, diabetes mellitus, current smoker, previous PCI, family history of CAD, creatinine, LVEF, ACC/AHA lesion classification, and IVUS use as covariates. The HR for CVA at 1 month could not be estimated because no events occurred in the non-MI group. P for interaction was obtained from a multivariable Cox model including an interaction term between MI status and follow-up period (before vs. after 1 month), and tests whether the effect of MI status on each outcome differed between the two periods; a single value is presented per outcome, shown on the corresponding row in the landmark block.

CD, cardiac death; MI, myocardial infarction; CVA, cerebrovascular accident; ST, stent thrombosis; RR, repeat revascularization; TVR, target vessel revascularization; TLR, target lesion revascularization; TVF, target vessel failure; TLF, target lesion failure; NACE, net adverse clinical events (composite of CD, MI, CVA, total bleeding, and definite or probable stent thrombosis); CI, confidence interval; HR, hazard ratio; PCI, percutaneous coronary intervention; CAD, coronary artery disease; LVEF, left ventricular ejection fraction; ACC, American College of Cardiology; AHA, American Heart Association; IVUS, intravascular ultrasound.

At 1 year, the cumulative incidence of NACE was numerically higher in the MI group than in the non-MI group, 20.5% vs. 14.9%, corresponding to a relative difference of approximately 40% without statistical significance (Table 4). Bleeding showed a similar borderline pattern (16.5% vs. 12.1%; HR 1.41, 95% CI 1.00–2.00; *p* = 0.053). The 1-year incidence of TLF was 6.0% in the overall study population (5.3% in the non-MI group and 7.2% in the MI group, *p* = 0.482), and 1-year TLR occurred in 3.3% of all patients (3.2% vs. 3.5%, *p* = 0.945). All other individual endpoints were comparable between the two groups.

**Table 4:** One-year clinical outcomes in MI versus non-MI patients.

Outcome	Total (*n* = 932)	Non-MI (*n* = 586)	MI (*n* = 346)	Log-rank *p*-value	Univariable HR (95% CI)	*p*-value	Multivariable HR (95% CI)	*p*-value
CD	25 (2.7)	12 (2.1)	13 (3.8)	0.150	1.78 (0.80–3.92)	0.156	0.79 (0.29–2.12)	0.636
MI	8 (0.9)	6 (1.0)	2 (0.6)	0.435	0.53 (0.11–2.65)	0.443	0.34 (0.06–1.95)	0.224
CVA	9 (1.0)	6 (1.0)	3 (0.9)	0.851	0.88 (0.22–3.50)	0.852	1.31 (0.27–6.30)	0.738
Total Bleeding	128 (13.7)	71 (12.1)	57 (16.5)	0.050	1.41 (1.00–2.00)	0.053	1.40 (0.93–2.10)	0.104
Major bleeding	8 (0.9)	5 (0.9)	3 (0.9)	0.927	–	–	–	–
Minor bleeding	120 (12.9)	66 (11.3)	54 (15.6)	0.045	–	–	–	–
ST	6 (0.6)	3 (0.5)	3 (0.9)	0.500	1.72 (0.35–8.54)	0.505	2.15 (0.35–13.03)	0.404
RR	75 (8.0)	48 (8.2)	27 (7.8)	0.743	0.92 (0.58–1.48)	0.743	0.86 (0.51–1.47)	0.589
TVR	38 (4.1)	24 (4.1)	14 (4.1)	0.847	0.94 (0.48–1.81)	0.848	1.02 (0.49–2.13)	0.959
TLR	31 (3.3)	19 (3.2)	12 (3.5)	0.945	1.03 (0.50–2.12)	0.945	0.92 (0.41–2.09)	0.847
TVF	67 (7.2)	39 (6.7)	28 (8.1)	0.770	1.08 (0.66–1.75)	0.769	0.77 (0.43–1.37)	0.374
TLF	56 (6.0)	31 (5.3)	25 (7.2)	0.482	1.21 (0.71–2.06)	0.483	0.73 (0.39–1.38)	0.333
NACE	158 (17.0)	87 (14.9)	71 (20.5)	0.056	1.35 (0.99–1.85)	0.060	1.23 (0.85–1.78)	0.274

Values are n (%).

Hazard ratios (HRs) and 95% confidence intervals (CIs) were calculated using univariable and multivariable Cox proportional hazards models, with the non-MI group as the reference. The multivariable model included age, sex, hypertension, diabetes mellitus, current smoker, previous PCI, family history of CAD, creatinine, LVEF, ACC/AHA lesion classification, and IVUS use as covariates.

CD, cardiac death; MI, myocardial infarction; CVA, cerebrovascular accident; ST, stent thrombosis; RR, repeat revascularization; TVR, target vessel revascularization; TLR, target lesion revascularization; TVF, target vessel failure; TLF, target lesion failure; NACE, net adverse clinical events (composite of CD, MI, CVA, total bleeding, and definite or probable stent thrombosis); CI, confidence interval; HR, hazard ratio; PCI, percutaneous coronary intervention; CAD, coronary artery disease; LVEF, left ventricular ejection fraction; ACC, American College of Cardiology; AHA, American Heart Association; IVUS, intravascular ultrasound.

In contrast to the short-term findings, in the 1-month landmark analysis evaluating events occurring between 1 month and 3 years after PCI among patients who were event-free at 1 month, no significant differences were observed between the MI and non-MI groups for any endpoint, including the primary composite of NACE (12.4% vs. 13.3%; HR 0.84, 95% CI 0.55–1.29; *p* = 0.425) (Table 3) (Figure 2). All individual components of NACE and secondary endpoints were similarly distributed between the two groups, with all hazard ratios crossing unity. The interaction between MI status and follow-up period was not significant for any endpoint (P for interaction = 0.588 for NACE) (Table 3).

**Figure 2:**
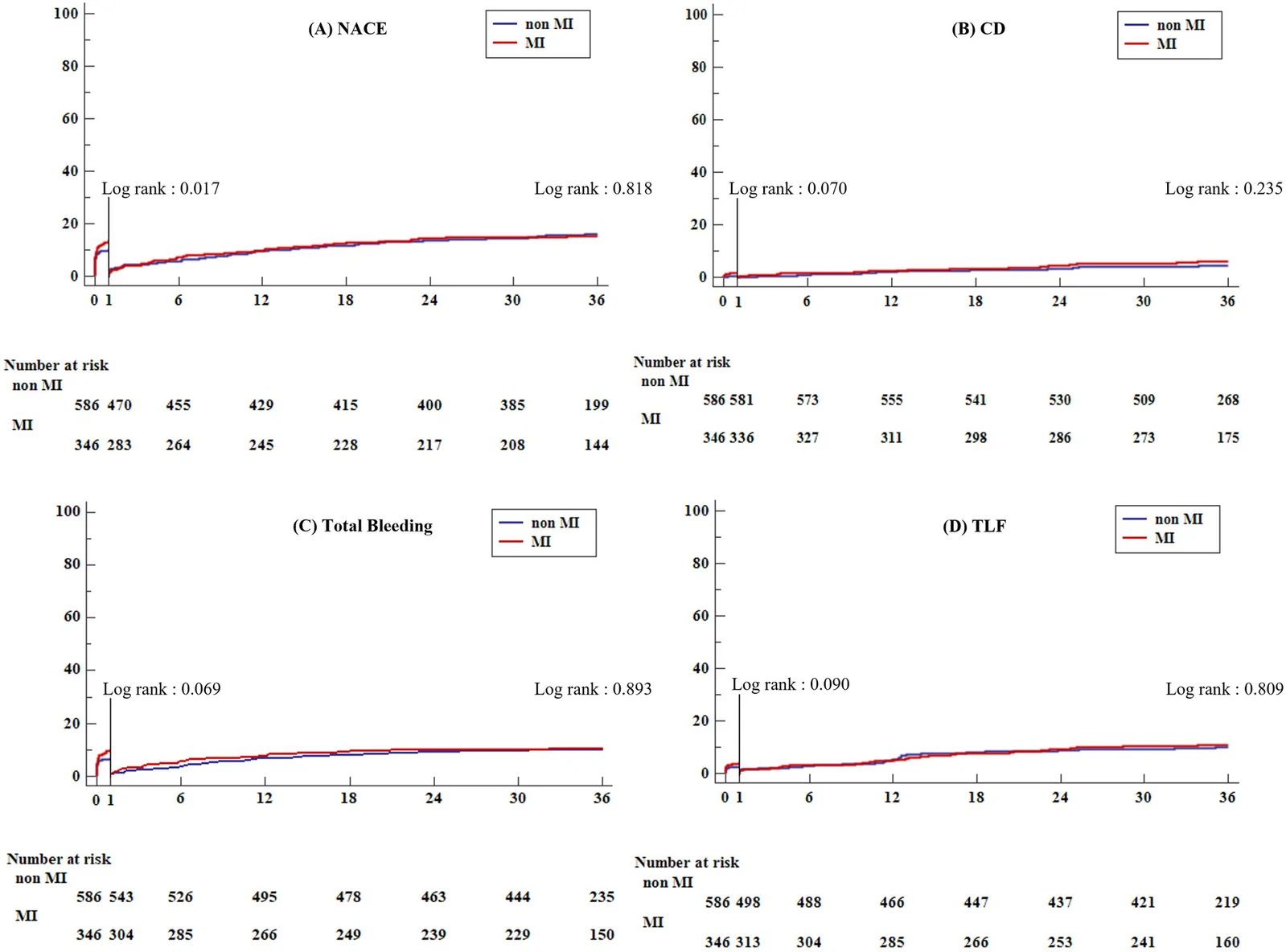
Kaplan–meier analysis of clinical outcomes during the 1-month landmark period kaplan–meier curves for the cumulative incidence of **(A)** net adverse clinical events (NACE), **(B)** cardiac death (CD), **(C)** total bleeding, and **(D)** target lesion failure (TLF) in MI versus non-MI patients. For each endpoint, outcomes are shown at 1 month and during the 1-month landmark period (from 1 month to 3 years among patients event-free at 1 month). Numbers at risk are shown at prespecified time points, and between-group differences were assessed using the log-rank test. MI = myocardial infarction; NACE = net adverse clinical events; CD = cardiac death; TLF = target lesion failure.

## Discussion

This prospective, multicenter Korean registry is the first to evaluate the clinical outcomes of patients undergoing PCI with the Ultimaster^TM^ BP-SES according to MI status across early and long-term follow-up, using a 1-month landmark approach. The main findings of this study are as follows. First, during the first month after PCI, the incidence of NACE was significantly higher in the MI group than in the non-MI group, with a numerical excess in CD and bleeding events. Second, the cumulative incidence of NACE at 1 year remained approximately 40% higher in the MI group, although this difference did not reach statistical significance, and the 1-year incidence of TLF and TLR was low and comparable to that reported in contemporary BP-DES registries. Third, in the 1-month landmark analysis evaluating events from 1 month to 3 years, no significant differences were observed between the MI and non-MI groups for any clinical endpoint, including the primary composite of NACE. Taken together, these findings indicate that the early excess of clinical events in patients with MI did not extend into the later, predominantly stent-related period of follow-up, which is reassuring with regard to the long-term safety and efficacy of BP-SES across the spectrum of clinical presentations. In a subgroup analysis by MI subtype, outcomes were comparable between STEMI and NSTEMI in both periods, including the stent-related endpoints during the landmark period (Supplementary Table S1). Despite their distinct thrombotic and occlusive characteristics, this similarity is consistent with the view that later stent-related outcomes are governed by the healing of the biodegradable-polymer stent, consistently across MI subtypes, whereas the early excess risk reflects the acute presentation common to both. Clinically, this suggests that long-term stent-related risk did not differ appreciably by MI subtype, and that management during the first month may be an important determinant of outcomes.

The baseline characteristics of our study were consistent with those reported in prior studies of patients with and without MI. Patients in the MI group were significantly younger and were more often men, current smokers, and individuals with a family history of coronary artery disease, whereas hypertension, diabetes mellitus, and previous PCI were less prevalent than in the non-MI group. A similar profile—including younger age, fewer comorbidities, and a higher proportion of smokers in patients presenting with acute MI—has been consistently reported in previous trials and long-term registries comparing acute MI with non-MI indications for PCI (9–11). The cardiovascular risk-factor profile of our MI subgroup—with hypertension in 51.7%, current smoking in 34.4%, and diabetes mellitus in 28.3%—closely paralleled the corresponding rates of 51.1%, 36.3%, and 30.0% reported by the nationwide Korea Acute Myocardial Infarction Registry for Regional Cardiocerebrovascular Centers (KAMIR-RCC) (15). In addition, total and LDL-C levels were higher and left ventricular ejection fraction was lower in the MI group, reflecting the more acute and higher-risk clinical profile of patients presenting with MI.

The 1-year incidence of TLF in our overall study was 6.0%, with a slightly higher rate in the MI group (7.2%) than in the non-MI group (5.3%). This rate was broadly comparable to those reported in previous BP-SES studies, while being at the upper end of the reported range. A previous Korean Multicenter Ultimaster Registry reported a 1-year TLF rate of 4.1% in an all-comers Korean population (8). and the multicontinental e-Ultimaster registry, the Italian ULISSE registry, and the CENTURY II trial reported 1-year TLF rates of approximately 3 to 5% across diverse populations (4, 6, 7). The slightly higher TLF rate in our study likely reflects the more complex coronary anatomy in our study [type C lesions in 75.1% vs. 62.5% in the previous Korean Ultimaster registry, an established predictor of TLF after contemporary DES (16)] and the higher proportion of acute coronary syndrome (ACS; 77.5% vs. 67.9%, including STEMI in 20.2% vs. 16.0%) (8).

The significantly higher early NACE in the MI group during the first month after PCI was driven primarily by a numerical excess in CD and bleeding events. The greater early CD is largely explained by patient- and infarction-related factors, including lower left ventricular ejection fraction and more complex coronary anatomy in our MI group, both of which are recognized predictors of adverse outcomes after primary PCI for acute MI (16, 17). The numerical excess in early bleeding events likely reflects differences in adjunctive antithrombotic therapy: while approximately 86% of non-MI patients received clopidogrel, about two-thirds of MI patients were discharged on potent P2Y12 inhibitors (ticagrelor or prasugrel), which may have contributed to the higher incidence of bleeding events in the MI group. These observations suggest that the early imbalance in clinical events between MI and non-MI patients reflects the inherent vulnerability of acute MI presentations and the more potent antiplatelet regimens used in this setting, rather than stent-related complications.

Although the cumulative incidence of NACE at 1 year remained approximately 40% higher in the MI group as a continued trend after the early excess at 1 month, all clinical endpoints from 1 month to 3 years—including the primary composite of NACE, individual components, and stent-related outcomes such as ST, TLF, TLR, and TVR—were comparable between the MI and non-MI groups in the 1-month landmark analysis. The interaction between MI status and follow-up period did not reach statistical significance for any endpoint (P for interaction = 0.588 for NACE), which may partly reflect the limited statistical power of interaction testing and the modest number of early events. A similar attenuation of early differences during long-term follow-up has been reported in previous studies of patients undergoing PCI with contemporary DES. In one such study, the 1-year incidence of death or MI, TLF, and ST was higher in patients presenting with ACS—a population that included a substantial proportion of MI—than in patients with stable coronary artery disease, but in the 1-year landmark analysis, no significant differences in clinical outcomes were observed between the two groups through 5 years (11). Similarly, in a pooled patient-level analysis of five randomised trials, patients with ACS, who again included a substantial proportion of MI, had a higher risk of mortality within the first year after PCI but comparable risk in the 1-year landmark analysis through 10 years, with no significant difference in TLR or TVR (18). Mechanistically, this convergence is consistent with the theoretical advantages of polymer resorption and improved vascular healing (19), particularly in vessels treated for acute MI, where arterial healing is known to be delayed, although a device-specific effect cannot be established in the absence of a comparator stent.

Improvements in patient care beyond stent design are likely to have contributed to this pattern as well. Secondary prevention in both groups reflected contemporary practice, with statins prescribed at discharge in more than 90% of patients and potent P2Y12 inhibitors and beta-blockers used more frequently in the MI group. Compared with the era of earlier studies (10, 20), the wider use of potent P2Y12 inhibitors and intensive lipid-lowering therapy after MI has lowered the risk of recurrent ischemic events, and this progress in medical therapy may have narrowed the long-term difference between MI and non-MI patients independently of the stent platform. The comparable long-term outcomes in this study should therefore be viewed as the combined result of a contemporary BP-SES and contemporary secondary prevention, rather than of the stent alone.

In contrast, an earlier substudy of a randomised trial reported that, at 18 months of cumulative follow-up, patients presenting with ACS showed a numerically higher rate of major adverse cardiac events (MACE) and significantly higher rates of CD and definite or probable ST than those treated for stable angina (20). The discrepancy with our findings likely reflects the use of older-generation durable-polymer stents in that trial, in which delayed endothelialisation may continue to drive late ischaemic events particularly in vessels treated for acute presentations, and the absence of an early-period landmark approach that, in our analysis, allowed the early excess of clinical events to be separated from the subsequent stent-related period; meta-analyses have since shown that contemporary BP-SES provide long-term safety and efficacy comparable to durable-polymer DES in patients with ACS (21). More recent large-scale evidence is consistent with our observations. In a randomised all-comers trial of 1,653 patients treated with contemporary DES, 5-year rates of target lesion failure, patient-oriented composite events, and stent thrombosis did not differ between patients presenting with acute and chronic coronary syndromes (22). Similarly, an individual patient data meta-analysis of randomised trials confirmed comparable 5-year outcomes across contemporary stent platforms in patients presenting with ACS (23). These studies, however, compared acute vs. chronic coronary syndromes as broad categories, without specifically isolating patients with myocardial infarction, and did not separate the early periprocedural phase from subsequent follow-up. By distinguishing patients with MI from those without and applying a 1-month landmark, the present study shows that the excess risk associated with MI is confined to the first month, after which outcomes are comparable through 3 years in routine clinical practice.

Taken together, the present findings suggest that, in the era of contemporary BP-SES and contemporary secondary prevention, MI status itself did not appear to be a major determinant of long-term clinical outcomes after PCI in this study, and that patients presenting with MI may expect outcomes similar to those of non-MI patients once the early high-risk period is past. From a clinical standpoint, this is reassuring for the use of BP-SES across the spectrum of clinical presentations, although these findings do not establish equivalence of long-term risk, and the absence of a comparator stent precludes conclusions regarding device-specific benefit.

Several limitations of this study should be acknowledged. First, this was an observational, non-randomized registry. Although enrollment was consecutive and all-comers, the possibility of selection bias inherent to a registry design cannot be entirely excluded. In addition, despite multivariable adjustment, residual confounding from unmeasured baseline differences between the MI and non-MI groups cannot be excluded. Second, our analysis was based on a single thin-strut bioabsorbable polymer sirolimus-eluting stent platform, and our findings may therefore not be generalizable to other contemporary biodegradable polymer or durable polymer drug-eluting stents. Third, angiographic lesion classification was based on operator assessment at each participating site, and independent core laboratory adjudication was not performed, which may have introduced subjective bias. However, the primary endpoint of this study was a composite of clinical events rather than angiographic measurements, and all clinical events were independently adjudicated by a centralized Clinical Events Committee. Fourth, routine angiographic follow-up was not performed and procedures were not stratified according to clinical urgency; therefore, subclinical restenosis may have been underestimated, and the differential impact of emergent vs. elective PCI could not be assessed. In addition, although a subgroup analysis by MI subtype was performed, the limited number of events precluded more detailed high-risk subgroup analyses. Finally, the study population was confined to Korean patients enrolled at tertiary cardiovascular centers, which may limit generalizability to other ethnic groups and clinical settings.

## Conclusion

In this prospective Korean registry, the early excess of clinical events observed in MI patients undergoing PCI with the Ultimaster^TM^ BP-SES did not extend beyond the first month, and no significant differences in outcomes through 3 years were detected compared with non-MI patients. These findings suggest that, in the era of contemporary BP-SES and contemporary secondary prevention, MI status itself does not appear to be a major determinant of long-term clinical outcomes after PCI, and are reassuring for the use of BP-SES across the spectrum of clinical presentations, although they do not establish equivalence of long-term risk or device-specific benefit. Larger comparative studies with longer follow-up are warranted to confirm these findings.
