# Supplementary material for: Impact of myocardial infarction status on long-term outcomes after PCI with the ultimaster biodegradable-polymer sirolimus-eluting stent
Source: Front Cardiovasc Med. 2026 Oct 30;13:1903710. doi: 10.3389/fcvm.2026.1903710 (PMC13760496; doi:10.3389/fcvm.2026.1903710)
Supplement: Supplementary file 1 [file Table1.docx]

**Supplementary Table 1. Clinical outcomes at 1 month and during the 1-month landmark period according to MI subtype (STEMI versus NSTEMI)**

| **Outcome** | **NSTEMI (n = 158)** | **STEMI (n = 188)** | **Log-rank p-value** | **Univariable HR (95% CI)** | **p-value** | **Multivariable HR (95% CI)** | **p-value** |
| --- | --- | --- | --- | --- | --- | --- | --- |
| ***At 1 month*** | | | | | | | |
| CD | 3 (1.9) | 2 (1.1) | 0.517 | 0.56 (0.09–3.34) | 0.523 | 0.82 (0.14–4.83) | 0.826 |
| MI | 0 (0.0) | 1 (0.5) | 0.356 | — | — | — | — |
| CVA | 1 (0.6) | 1 (0.5) | 0.893 | 0.83 (0.05–13.23) | 0.893 | 1.53 (0.09–24.80) | 0.767 |
| Total Bleeding | 14 (8.9) | 19 (10.1) | 0.706 | 1.14 (0.57–2.27) | 0.710 | 1.33 (0.66–2.67) | 0.420 |
| ST | 0 (0.0) | 2 (1.1) | 0.194 | — | — | — | — |
| RR | 0 (0.0) | 3 (1.6) | 0.111 | — | — | — | — |
| TVR | 0 (0.0) | 3 (1.6) | 0.111 | — | — | — | — |
| TLR | 0 (0.0) | 3 (1.6) | 0.111 | — | — | — | — |
| TVF | 3 (1.9) | 6 (3.2) | 0.450 | 1.69 (0.42–6.77) | 0.457 | 1.82 (0.46–7.24) | 0.396 |
| TLF | 3 (1.9) | 6 (3.2) | 0.450 | 1.69 (0.42–6.77) | 0.457 | 1.82 (0.46–7.24) | 0.396 |
| NACE | 18 (11.4) | 24 (12.8) | 0.694 | 1.13 (0.61–2.08) | 0.700 | 1.32 (0.71–2.44) | 0.380 |
| ***From 1 month to 3 years (landmark analysis)*** | | | | | | | |
| CD | 12 (7.6) | 8 (4.3) | 0.240 | 0.59 (0.24–1.44) | 0.246 | 0.87 (0.35–2.19) | 0.774 |
| MI | 1 (0.6) | 1 (0.5) | 0.910 | 0.85 (0.05–13.63) | 0.910 | 0.91 (0.06–14.88) | 0.945 |
| CVA | 1 (0.6) | 4 (2.1) | 0.244 | 3.41 (0.38–30.48) | 0.273 | 3.27 (0.43–25.13) | 0.255 |
| Total Bleeding | 13 (8.2) | 16 (8.5) | 0.922 | 1.04 (0.50–2.16) | 0.922 | 1.08 (0.51–2.26) | 0.847 |
| ST | 0 (0.0) | 1 (0.5) | 0.353 | — | — | — | — |
| RR | 20 (12.7) | 15 (8.0) | 0.175 | 0.63 (0.32–1.23) | 0.179 | 0.66 (0.34–1.30) | 0.228 |
| TVR | 10 (6.3) | 6 (3.2) | 0.193 | 0.52 (0.19–1.42) | 0.201 | 0.53 (0.19–1.47) | 0.220 |
| TLR | 7 (4.4) | 5 (2.7) | 0.404 | 0.62 (0.20–1.94) | 0.409 | 0.58 (0.18–1.85) | 0.357 |
| TVF | 22 (13.9) | 13 (6.9) | 0.046 | 0.50 (0.25–1.00) | 0.050 | 0.60 (0.30–1.20) | 0.150 |
| TLF | 20 (12.7) | 12 (6.4) | 0.061 | 0.51 (0.25–1.05) | 0.066 | 0.60 (0.29–1.23) | 0.159 |
| NACE | 22 (13.9) | 21 (11.2) | 0.510 | 0.82 (0.45–1.49) | 0.511 | 0.94 (0.51–1.73) | 0.849 |

Values are n (%).

Hazard ratios (HRs) and 95% confidence intervals (CIs) were calculated using univariable and multivariable Cox proportional hazards models, with the NSTEMI group as the reference. The multivariable model included age, sex, hypertension, diabetes mellitus, current smoker, previous PCI, family history of CAD, creatinine, LVEF, ACC/AHA lesion classification, and IVUS use as covariates. HRs could not be estimated for outcomes with no events in the NSTEMI group. Given the small number of events within each subtype, these subgroup analyses should be regarded as exploratory.

STEMI, ST-segment elevation myocardial infarction; NSTEMI, non–ST-segment elevation myocardial infarction; MI, myocardial infarction; CD, cardiac death; CVA, cerebrovascular accident; ST, stent thrombosis; RR, repeat revascularization; TVR, target vessel revascularization; TLR, target lesion revascularization; TVF, target vessel failure; TLF, target lesion failure; NACE, net adverse clinical events (composite of CD, MI, CVA, total bleeding, and definite or probable stent thrombosis); CI, confidence interval; HR, hazard ratio; PCI, percutaneous coronary intervention; CAD, coronary artery disease; LVEF, left ventricular ejection fraction; ACC, American College of Cardiology; AHA, American Heart Association; IVUS, intravascular ultrasound.
